# Polypoid arteriovenous malformation of the rectum: A case report

**DOI:** 10.3389/fsurg.2022.924801

**Published:** 2022-07-14

**Authors:** Dimitri Krizzuk, Maria Cotesta, Giampaolo Galiffa, Ilaria Peluso, Francesco Falbo, Andrea Biancucci, Sara Puscio, Chiara Michelotto, Carolina Pasecinic, Gioacchino Maria Montalto, Francesco Sammartino

**Affiliations:** ^1^ Department of General and Minimally-Invasive surgery, Aurelia Hospital, Rome, Italy; ^2^ Department of general surgery, Policlinico Tor-Vergata University, Rome, Italy

**Keywords:** intestinal arteriovenous malformation, polypoid arteriovenous malformation of the rectum, rectal bleeding, laparoscopic rectal resection, rectal polyp

## Abstract

**Background:**

Intestinal arteriovenous malformation is an abnormal connection between arteries and veins that bypasses the capillary system and may be a cause of significant lower gastrointestinal bleeding. On endoscopy, arteriovenous malformations are usually flat or elevated, bright red lesions. Overall, rectal localization of arteriovenous malformations is rare. The same may be said about polypoid shape arteriovenous malformations. Herein, we present a case of a large rectal polypoid arteriovenous malformations.

**Methods:**

Clinical, diagnostic, and treatment modalities of the patient were reviewed. Pre- and post-operative parameters were collected and analyzed. The clinical English literature is also reviewed and discussed

**Results:**

A 60-year-old female patient was admitted to our emergency department for rectorrhagia and anemia. Rectoscopy revealed a polypoid lesion in the rectum and the biopsy showed fibrosis, necrosis areas, and hyperplastic glands. A total body contrast-enhanced computed tomography (CT) was performed revealing a parietal pseudonodular thickening with concentric growth and contrast enhancement, extending for about 53 mm. The mass wasn't removed endoscopically due to concentric growth, sessile implant, and submucosal nature. The patient underwent an uneventful laparoscopic anterior rectal resection. The postoperative hospitalization was free of complications. Histology showed the presence of a polypoid AVM composed of dilated arteries, veins, capillaries, and lymphatics, engaging the submucosa, muscularis, and subserosa layer.

**Conclusion:**

After a review of the current English literature, we found only one case of rectal polypoid AVM. The scarcity of documented cases encumbers optimal diagnostic and treatment approaches.

## Introduction

Intestinal Arteriovenous malformation (AVM) is a cause of lower gastrointestinal bleeding alongside diverticula, colon neoplasia, and internal hemorrhoids (1). AVM is an abnormal connection between arteries and veins that bypasses the capillary system (2). Typical symptoms of intestinal AVMs are intermittent bloody stools without abdominal pain and anemia (3). Generally, on endoscopy, AVM appears as a flat or elevated, bright red lesion (4). The polypoid shape is extremely rare and only a few cases are described in the literature. Moreover, gastrointestinal AVMs are mostly localized in the right colon (37%) and small intestine (43%) but rarely in the rectum (8%) (2). To the best of our knowledge, only one case of rectal polypoid AVM was previously documented in the literature (5). No treatment guideline exists, and the uniqueness of this condition may lead to misdiagnosed cases and suboptimal care. Herein is a presentation of a case of a 53 mm polypoid shape AVM of the rectum along with a discussion of the available literature. This article aims to discuss only polypoid rectal AVMs and other vascular lesions such as vascular ectasia/angiodysplasia, cavernous hemangioma, and AVM located in other sections of the gastrointestinal tract, will not be a part of the analysis.

## Case report

A sixty-year-old female patient was admitted to our emergency department for rectorrhagia and anemia. Her medical history was significant for chronic atrial fibrillation treated with rivaroxaban and propafenone. On arrival, she was hemodynamically stable, and the level of hemoglobin was 9.6 g/dl. Other laboratory tests were not remarkable. Digital rectal exploration was performed showing circumferential hemorrhoids without bleeding. For further investigation, rectoscopy was accomplished and revealed an intraluminal, submucosal, polypoid lesion in the rectum. The mass was biopsied, and the histology showed fibrosis, necrosis areas, and hyperplastic glands. The patient was admitted to General Surgery Department to complete the clinical assessment.

**Figure 1:**
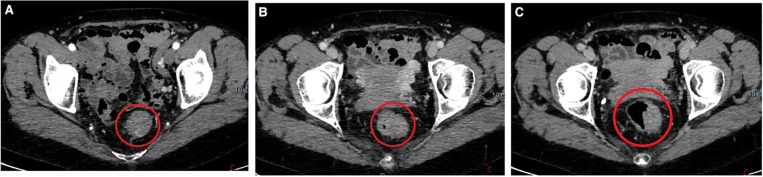
(**A**) contrast enhanced computer tomography, arterial phase. Red circle marks the rectum. (**B**) Contrast enhanced computer tomography, portal phase. Red circle marks the rectum, (**C**) Contrast enhanced computer tomography, nephrogenic phase. Red circle marks the rectum.

**Figure 2:**
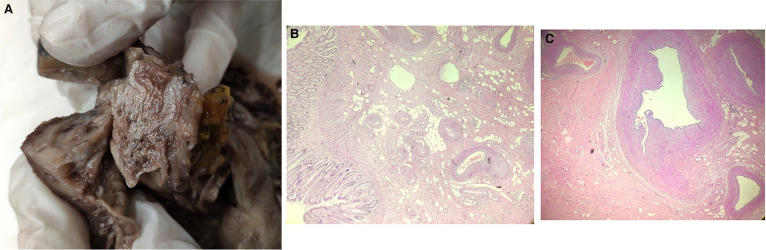
(**A**) macroscopic appearance of the polypoid AVM after fixation. (**B**) hematoxylin and eosin large field magnification of the polypoid rectal AVM. (**C**) hematoxylin and eosin small field magnification of the polypoid rectal AVM.

A total body contrast-enhanced computed tomography (CT) was performed revealing a parietal pseudonodular thickening with concentric growth and contrast enhancement, extending for about 53 mm (Figure 1). Rounded lymph nodes were spotted in the perirectal tissue. A completion colonoscopy was accomplished confirming a hemorrhagic submucosal polypoid lesion with concentric growth in the rectum, localized at 10 cm from the anal verge. The mass wasn't removed endoscopically due to concentric growth, sessile implant, and submucosal nature. Furthermore, the rounded lymph nodes in perirectal tissue were deemed suspicious, and the lesion was distally tattooed in the anticipation of surgical resection. A diagnosis of Gastro-Intestinal Stromal Tumor (GIST) was assumed based on the clinical, macroscopic, and radiologic appearance. Due to the resectability of the nodule, no fine needle biopsy was made, and no further diagnostics were deemed to be necessary (6). An indication for surgical resection was given and the patient underwent an uneventful laparoscopic anterior rectal resection. The postoperative hospitalization was free of complications, and the patient was discharged on the 5th postoperative day. Histology showed presence of a polypoid AVM composed of dilated arteries, veins, capillaries, and lymphatics, engaging the submucosa, muscolaris and subserosa layer. Chronic inflammation and mucosal ulceration were present (Figure 2). Thirteen negative lymph nodes were harvested. No 30-day complications or readmission were observed and at 12 months follow-up the patient was well. Her hemoglobin level stabilized at 13.7 g/dl.

## Discussion

Gastrointestinal tract AVMs are a rare disease and are reported to account for 0.8%–3% of all cases of intestinal bleeding (7). Various vascular malformations of the bowel are described, mainly: vascular ectasia/angiodysplasia, cavernous hemangioma, and AVM. All these conditions are bonded together by the presence of abnormal vessels in the bowel wall, but each has peculiarities that make them unique in clinical presentation, appearance, and management. The scope of this article is to report a rare case of polypoid rectal AVM. The above-mentioned vascular lesions are not a part of this analysis and will not be discussed.

AVMs are extremely rare in people under the age of 50 (1). The pathophysiology of AVMs is uncertain. It may be caused by intermittent, low-grade ischemia due to the obstruction of submucosal veins penetrating the muscularis or depend on acquired vascular degenerative disease associated with aging. These phenomena are thought to lead to the development of these abnormal arteriovenous communications (8). Typically, AVMs appear as a flat, bright red lesion and they rarely occur in polypoid shape (4). Lesions are mainly located in the right colon (37%), followed by the jejunum (24%) and ileus (19%). Overall, rectal, flat, and polypoid lesions account for only 8% (2). Typical symptoms of intestinal AVMs are intermittent, painless bloody stools, and anemia (3). Histopathologically, AVMs of the gastrointestinal tract are characterized by widespread full-thickness vasodilation from the submucosa to the serosa and are composed of an inflow artery (feeder), an abnormal blood vessel assembly (nidus), and an outflow vein (drainer) (2, 9).

*Moore et al.* reviewed and classified intestinal AVMs based on angiographic characteristics, localization, patient age, and family history (10). The authors classified AVMs into 3 types. Type 1 AVMs are solitary, small (usually <5 mm), localized mostly in the right colon, and typically seen in elderly patients. Type 2 AVMs are typically large lesions seen in the small intestine and can be a source of obscure GI bleeding. They are considered congenital. Type 3 AVMs are punctuate angiomas that commonly cause GI hemorrhage (10, 11). Some authors suggest that polypoid lesions, as seen in our case, shouldn't be classified based on *Moore et al*. classification, because of distinct clinical and endoscopic characteristics and different management strategies (1, 4). In fact, in the present case, the AVM does not fit any of the 3 categories and probably can't be classified with the use of the Moore classification.

Diagnosis of intestinal AVMs is based on colonoscopy, contrast-enhanced CT, and MRI. In cases of active bleeding, accurate localization of the source of gastrointestinal hemorrhage is an important factor in determining appropriate management. Polypoid rectal AVM, due to its submucosal localization and rarity, presents a diagnostical challenge. This, as in our case, may lead to misdiagnosis and subsequent suboptimal treatment or intra- and post-operative complications.

Various AVM treatment possibilities were suggested. Generally speaking, AVMs can be treated with endoscopic hemostasis and resection, intravascular embolization, and surgery (2, 12). Minimally invasive procedures are preferable in bad surgical candidates (2, 12). Polypoid AVMs are usually treated endoscopically. In a recent paper, Rzepczynski et al. stated that only 15 cases of colonic polypoid AVMs were reported in the English literature. Only one case was treated surgically, while the remaining 14 cases underwent an endoscopic resection (4). Maeng et al. described another surgically treated, 6 cm large, polypoid AVM of the transverse colon (9). The only case of rectal polypoid AVM was described by McKevitt et al. and consisted of a 7 mm lesion causing hematochezia in a 24-year-old male. It was treated by hemostasis with epinephrine injection and a subsequent repeat sigmoidoscopy with snare resection and cauterization (5). Due to its rareness, there is no standard treatment protocol or surgical procedure for rectal AVM, particularly if of large dimensions. Our case shows a 53 mm sessile polypoid AVM of the proximal rectum causing rectal bleeding with anemia and treated with laparoscopic anterior rectal resection and proximal total mesorectal excision (pTME). Examination of the current literature indicates that such treatment is adequate and may be indicated in cases of large polypoid AVMs of the rectum as it is for large rectal hemangiomas (13). In our opinion, minimally invasive natural orifice techniques such as transanal minimally invasive surgery (TAMIS) or Endoscopic full-thickness resection (EFTR), should be explored as valid alternatives to laparoscopic rectal resection.

## Conclusion

Optimal, evidence-based management of AVMs is challenging due to the paucity of literature on the subject, even more so, the management of rectal polypoid AVMs, of which only one case is reported to date. Small polypoid AVM lesions can be safely treated endoscopically. Surgical resection may be indicated for larger AVMs. Due to the scarcity of documented cases, no strong recommendations may be given on the treatment modality. Reporting other cases may aid in the guidance of therapeutic approach.

## Data Availability

The original contributions presented in the study are included in the article/Suplementary Material, further inquiries can be directed to the corresponding author/s.
